# Do We Need Intraoperative Magnetic Resonance Imaging in All Endoscopic Endonasal Pituitary Adenoma Surgery Cases? A Retrospective Study

**DOI:** 10.3389/fonc.2021.733838

**Published:** 2021-10-01

**Authors:** Emrah Celtikci, Muammer Melih Sahin, Mustafa Caglar Sahin, Emetullah Cindil, Zuhal Demirtaş, Hakan Emmez

**Affiliations:** ^1^ Department of Neurosurgery, Gazi University Medical School, Ankara, Turkey; ^2^ Department of Otorhinolaryngology, Gazi University Medical School, Ankara, Turkey; ^3^ Department of Radiology, Gazi University Medical School, Ankara, Turkey

**Keywords:** adenoma, endoscopy, intraoperative, magnetic resonance, pituitary

## Abstract

There are previous reports investigating effectiveness of intraoperative magnetic resonance imaging (IO-MRI) in pituitary adenoma surgery but there is no clear data in the literature recommending when there is no need of intraoperative scan. This retrospective analysis was based on determining which patients does not need any IO-MRI scan following endoscopic endonasal pituitary adenoma surgery. Patients with functional or non-functional pituitary adenomas that were operated *via* endoscopic endonasal approach (EEA) between June 2017 and May 2019 were enrolled. Patients younger than 18 years old, patients who did not underwent IO-MRI procedure or not operated *via* EEA were excluded from the study. Hence, this study is designed to clarify if IO-MRI is useful in both functional and non-functional pituitary adenomas, functional adenomas did not split into subgroups. A total of 200 patients treated with pituitary adenoma were included. In Knosp Grade 0 – 2 group, primary surgeon’s opinion and IO-MRI findings were compatible in 150 patients (98.6%). In Knosp Grade 3 – 4 correct prediction were performed in 32 (66.6%) patients. When incorrectly predicted Knosp Grade 3 – 4 patients (n = 16) was analyzed, in 13 patients there were still residual tumor in cavernous sinus and in 3 patients there were no residual tumor. Fisher’s exact test showed there is a statistically significant difference of correct prediction between two different Knosp Grade groups (two-tailed P < 0.0001). Eighteen patients had a residual tumor extending to the suprasellar and parasellar regions which second most common site for residual tumor. Our findings demonstrate that there is no need of IO-MRI scan while operating adenomas limited in the sellae and not invading the cavernous sinus. However, we strongly recommend IO-MRI if there is any suprasellar and parasellar extension and/or cavernous sinus invasion.

## Introduction

Endoscopic endonasal approach (EEA) to the sellar region provides wide panoramic view during pituitary adenoma surgery allowing visualization of far lateral parts of the surgical field which is not possible with microscopic transsphenoidal surgery. Recent studies demonstrated superiority of EEA in resection of pituitary adenomas (1). Even to the advances in the endoscopy technology, gross total resection rates are not more than 80% in large series (2–9).

Following the first report by Jolesz and Blumenfeld, intraoperative magnetic resonance imaging (IO-MRI) became popular all over the world (10). Expected benefit of this MRI system in pituitary adenoma surgery is achieving higher rates of gross total resection (GTR) however, there are also controversies over higher costs and increased surgical time (11–21).

Here we present a study comprising 200 patients who underwent EEA pituitary adenoma resection by using IO-MRI. This retrospective analysis was based on the hypothesis, IO-MRI scan is not necessary during EEA in a particular group of patients. We aimed to clarify when we need IO-MRI scan and when we do not.

## Materials and Methods

### Patients

Patients with functional or non-functional pituitary tumors that were operated *via* EEA between June 2017 and May 2019 in Gazi University Faculty of Medicine Department of Neurosurgery were enrolled. The Institutional Review Board and Ethics Committee approved this retrospective study; oral and written consents of the patients were obtained prior to the study. Also, all patients were informed about the technique and gave their signed consent to IO-MRI during surgery and to the data being used for research purpose. Patients younger than 18 years old, patients who did not underwent IO-MRI procedure or not operated *via* EEA were excluded from the study. Hence, this study is designed to clarify if IO-MRI is useful in both functional and non-functional pituitary adenomas, functional adenomas did not split into subgroups.

### Technique

All patients were screened to exclude any contraindication to magnetic resonance imaging (MRI). All data were acquired using a 3-Tesla Magnetom Verio^®^ (Siemens, Erlangen, Germany). Intraoperative scans were performed with 8−channel 2−part coils and compatible head holders (NORAS MRI products GmbH, Hochberg, Germany). EEA was performed using skull base neurosurgical endoscopic instruments, including 4-mm rod-lens endoscopes (0°, 30° and 45°) that were coupled to a high-definition camera and an AIDA HD system (Karl Storz GmbH and Co.). Four-hand technique was used, both surgeons were neurosurgeons. Medial or lateral cavernous approaches were performed when needed. Prior performing IO-MRI scan, primary surgeon declared and noted his decision if he thinks there is residual tumor or not. Following IO-MRI scan, if there is a residual tumor primary surgeon decided for further removal of tumor or not. All patients underwent a final post-operative MRI scan when the senior surgeon decided not to continue the surgery. Cavernous sinus invasion was classified according to Knosp Grading (22).

### Complications

Postoperative meningitis was defined when antibiotic treatment was required because of clinical signs of meningeal inflammation even if no pathogen was isolated. Cerebrospinal fluid (CSF) fistula was considered as a complication if a lumbar drain or revision surgery was necessary. Furthermore, intraoperative and postoperative bleeding and new transient or permanent neurologic deficits were included.

### Data Analysis

Analysis of the retrospective data was performed using SPSS 21.0 (IBM Corp., Armonk, New York, USA). In order to compare predictions and to compare residual mass ratios between two Knosp Grade groups, Fisher’s exact test were used. Variables were; preoperative largest diameter of tumor (< 4 cm or ≥ 4 cm), Knosp grade, age, gender, and recurrent surgery.

## Results

### Patient and Tumor Characteristics

A total of 200 patients treated with pituitary adenoma were included. A mean age of 57.4 years (range, 19 – 75 years) was noted. 105 patients were male (52.5%). Eleven patients who had a history of previous adenoma surgery were operated for recurrent or residual adenoma. Patient and tumor characteristics are summarized in **Table 1**.

**Table 1:** Demographic and tumor characteristics of 200 patients underwent endoscopic endonasal resection of pituitary adenoma with intraoperative magnetic resonance imaging.

Characteristic	Value
**Age (years)**	
Median	57.4
Range	19 – 75
**Gender**	
Male	105
Female**/**Male ratio	1/1.05
**Tumor Volume and Cavernous Extension**	
Knosp Grade 3 – 4	48
Knosp Grade 0 – 2	152
Giant adenoma (≥ 4 cm in largest diameter)	31
**Hormonal Activity**	
Non-functional	94
**History of Pituitary Adenoma Surgery**	
Recurrent Surgery	28

Values other than ratios or patient age are number of patients.

### Knosp Grade and Hormonal Activity

Twenty-two patients had a Knosp Grade 3 – 4 functional pituitary adenoma. Following IO-MRI residual adenoma was detected in 9 (40.9%) patients and further resection is performed in all of them (100%). In 4 patients, in order to avoid complications, primary surgeon decided not to perform anymore residual tumor removal from cavernous sinus after the second IO-MRI scan. Twenty-six patients had a Knosp Grade 3 – 4 non-functional pituitary adenoma. Following IO-MRI residual adenoma was detected in 16 (61.5%) patients and further resection is performed in 3 (18.7%) of them. In all Knosp Grade 3 – 4 patients.

Following IO-MRI scan, 9 of 84 (10.7%) and 13 of 68 (19.1%) functional and non-functional residual adenoma was detected in Knosp Grade 0 – 2 group respectively. All Knosp Grade 0 – 2 patients who has a residual adenoma in both functional and non-functional groups underwent further removal of tumor. Tumor characteristics according to Knosp Grade is summarized in **Table 2**.

**Table 2:** Percentage and distribution of residual adenoma finding in first intraoperative magnetic resonance imaging according to tumor characteristic and Knosp Grade.

Tumor Characteristic	Knosp Grade		two-tailed P value
	0 – 2 (n = 152)	3 – 4 (n = 48)	
*Functional*	10.7% (9)	40.9% (9)	
*Non-functional*	19.1% (13)	61.5% (16)	
Total	14.4% (22)	52% (25)	< 0.001

Values are percentages of patients and number of patients are in parenthesis.

### Primary Surgeon Opinion and IO-MRI

In Knosp Grade 0 – 2 group, primary surgeon’s opinion and IO-MRI findings were compatible in 150 patients (98.6%). Both two patients which were incorrectly predicted by primary surgeon had giant adenomas and had residual tumor on IO-MRI scan. In Knosp Grade 3 – 4 correct prediction were performed in 32 (66.6%) patients. When incorrectly predicted Knosp Grade 3 – 4 patients (n = 16) was analyzed, in 13 patients there were still residual tumor in cavernous sinus and in 3 patients there were no residual tumor. Fisher’s exact test showed there is a statistically significant difference of correct prediction between two different Knosp Grade groups (two-tailed P < 0.0001) (**Table 3**).

**Table 3:** Comparison of primary surgeon opinion before intraoperative magnetic resonance imaging and findings after scan.

Opinion before IO-MRI	Knosp Grade				two-tailed P value
	0 – 2		3 – 4		
	No residual mass	Residual mass	No residual mass	Residual mass	
Correct	143	7	19	13	
Incorrect	0	2	13	3	< 0.0001

Values are number of patients.

### Complications

One patient (0.5%) had post-operative meningitis signs without any isolated pathogen. Two patients who had Knosp Grade 3 – 4 adenomas had ICA injury (1%), both on cavernous segment of the right ICA. Two patients had post-operative unilateral oculomotor nerve paralysis. In both patients IO-MRI showed residual tumor in the cavernous sinus and further resection was performed following scan. After 3-months follow up oculomotor function recovered completely in both patients. Eighteen patients had CSF fistula (9%) 15 of them treated with lumbar drain and 3 of them underwent revision surgery.

### Additional Findings

**Figure 1:**
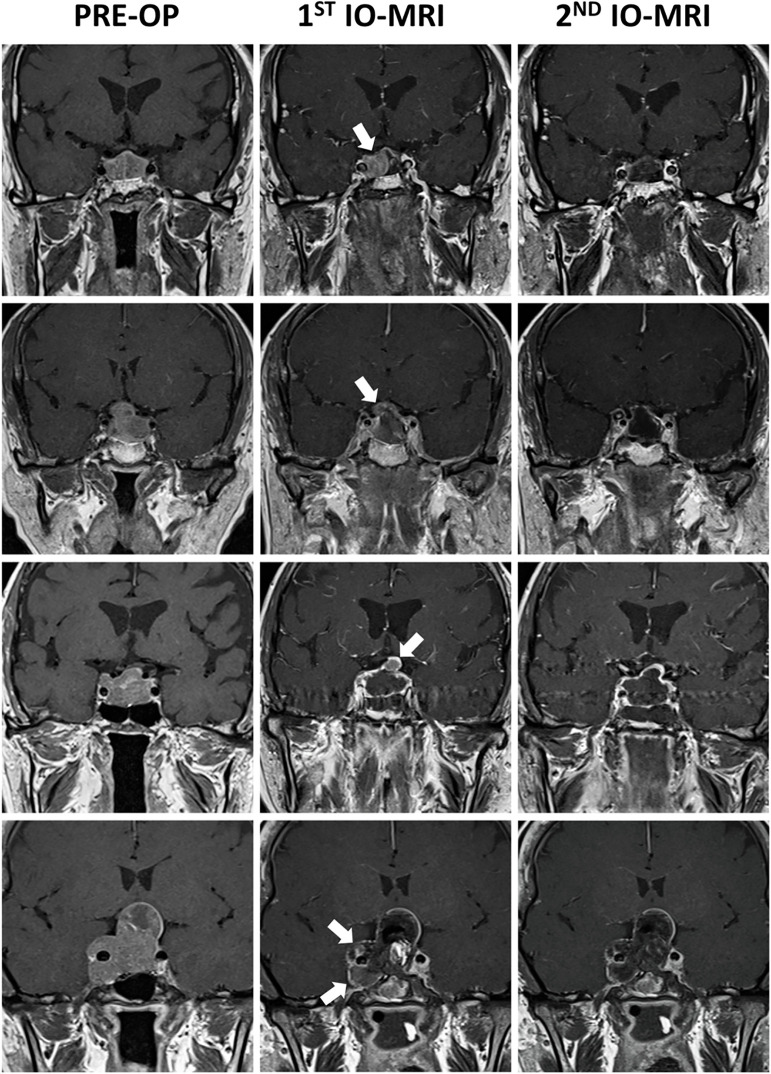
Figure demonstrating pre-operative and intraoperative contrast enhanced coronal T1-weighted pituitary MRIs of 4 different patients. First patient (first row) had a prolactin secreting macroadenoma which had two different signal intensity on pre-operative MRI scan without any cavernous invasion. Right side of the tumor was showing high signal intensity. First IO-MRI scan demonstrating residual mass on the right side. Second IO-MRI scan demonstrating that total removal was achieved. Second patient (second row) had a non-functioning macroadenoma causing visual disturbance. First IO-MRI scan demonstrating residual mass where tumor was penetrating the sellar diaphragm. Surgeon decided further excision and total removal was achieved. Third patient (third row) had a Knosp Grade 3A growth hormone secreting macroadenoma. Following excision, surgeon’s decision was in favor of total removal. However, first IO-MRI scan showed residual tumor was not in the cavernous sinus but in the region where tumor was penetrating the sellar diaphragm. Total removal was achieved following further excision. Last patient (fourth row) had a Knosp Grade 4 non-functioning pituitary adenoma causing severe visual disturbances. Following excision first IO-MRI demonstrated residual mass in the cavernous sinus. Further excision was performed which resulted in total removal of the tumor. Patient had a 3^rd^ nerve palsy following the surgery which totally improved after 3 months post-operatively. White arrows demonstrating residual tumor tissues on IO-MRIs.

Retrospective analysis of the patients’ scans revealed second most common site for residual tumor following cavernous sinus was suprasellar and parasellar extension. Eighteen patients (9%) had a residual tumor extending to the suprasellar and parasellar regions (**Figure 1**).

## Discussion

In this study we demonstrate that IO-MRI scan during EEA for resection of pituitary adenomas is not necessary for all tumor types. Our findings are showing that IO-MRI is beneficial in adenomas with suprasellar and parasellar extension and/or invading cavernous sinus (Knosp Grade 3 & 4). We found no additional benefit of IO-MRI in microadenomas, adenomas with no cavernous sinus invasion or adenomas limited in the sellae without any suprasellar and parasellar extension.

Endoscopic surgery of the pituitary adenomas became preferred treatment method in time by providing a viable median corridor and improved visualization of the anatomical landmarks (2, 23). Even to the technological advances, recurrence rates are up to 20% in the literature and first surgery is the most important intervention (24, 25). As a result, all efforts should be made to achieve total resection in the first surgery. One of the important factors preventing total resection of pituitary adenomas is cavernous sinus invasion. Following reevaluation of their MRI-based classification, Knosp et al. demonstrated that resection rates at the superior compartment of the cavernous sinus were higher than those at the inferior one (22, 26). Hence, cavernous sinus is on the agenda of neurosurgeons performing EEA, there are recent reports defining anatomical relationships and approaches to the cavernous sinus (3, 27–29). For this reason, we designed our retrospective study to clarify if IO-MRI scans were necessary when there is no significant cavernous sinus invasion or is there any pre-operative indicator to determine which patients will benefit from IO-MRI scans.

Other than our findings defining benefit of IO-MRI in resection of invasive adenomas, parasellar or suprasellar extension took our attention. Ramm-Pettersen et al. emphasized importance of IO-MRI when there is a parasellar or suprasellar extension of the adenoma (19). We agree that utilization of IO-MRI in endoscopic endonasal pituitary adenoma surgery improves resection rates when there is parasellar or suprasellar extension.

There are previous reports investigating effectiveness of IO-MRI in pituitary adenoma surgery but there is no clear data in the literature recommending when there is no need of intraoperative scan (14, 16, 17, 19–21). Previous studies are focusing effects of IO-MRI on surgical time, cost effectiveness, GTR and recurrence rates. On the other hand, we focused on to determine which patients does not need any IO-MRI scan. We believe our findings will guide neurosurgeons especially in the countries where MRI scans are costly.

There are limitations of this study. First, this is a retrospective study and tries to correlate results with only using MRI data. Study does not include and correlate any long-term follow up data including progression free survival rates. However, one of the aims of this study was to determine if there is any correlation between prediction of the surgeon and MRI findings. Additionally, we did not perform any cost-effectiveness analysis because study was performed in a country which has a universal health care plan for all the citizens and costs of MRI scans are relatively cheap (about 15 U.S. dollars per scan). This is one of the reasons why authors performed IO-MRI scans for all the patients in the study cohort.

Even though our findings are demonstrating IO-MRI does not have additional benefit in microadenomas, adenomas with no cavernous sinus invasion or adenomas limited in the sellae, we will continue to collect and analyze data *via* performing intraoperative scans in every patient undergoing EEA for pituitary adenomas. We are planning to determine effects of IO-MRI in endoscopic surgery of pituitary adenoma subtypes.

Utilization of IO-MRI in endoscopic endonasal pituitary adenoma surgery is increasing but current literature is focusing on surgical time, cost effectiveness, GTR and recurrence rates. Our findings demonstrate that there is no need of IO-MRI scan while operating adenomas limited in the sellae and not invading the cavernous sinus. However, we strongly recommend IO-MRI if there is any suprasellar and parasellar extension and/or cavernous sinus invasion.

## Data Availability Statement

The original contributions presented in the study are included in the article/supplementary material. Further inquiries can be directed to the corresponding author.

## Ethics Statement

The studies involving human participants were reviewed and approved by The Institutional Review Board and Ethics Committee. The patients/participants provided their written informed consent to participate in this study.

## Author Contributions

ECe and ZD wrote the paper. HE, MMS, and MCS edited the paper. ECi analyzed the IO-MRI. All authors contributed to the article and approved the submitted version.

## Conflict of Interest

The authors declare that the research was conducted in the absence of any commercial or financial relationships that could be construed as a potential conflict of interest.

## Publisher’s Note

All claims expressed in this article are solely those of the authors and do not necessarily represent those of their affiliated organizations, or those of the publisher, the editors and the reviewers. Any product that may be evaluated in this article, or claim that may be made by its manufacturer, is not guaranteed or endorsed by the publisher.

## References

[1] Almutairi RD Muskens IS Cote DJ Dijkman MD Kavouridis VK Crocker E. Gross Total Resection of Pituitary Adenomas After Endoscopic *vs*. Microscopic Transsphenoidal Surgery: A Meta-Analysis. Acta Neurochir (Wien) (2018) 160:1005–21. doi: 10.1007/s00701-017-3438-z PMC5899014 29307020

[2] Cappabianca P Cavallo LM Colao A de Divitiis E. Surgical Complications Associated With the Endoscopic Endonasal Transsphenoidal Approach for Pituitary Adenomas. J Neurosurg (2002) 97:293–8. doi: 10.3171/jns.2002.97.2.0293 12186456

[3] Fernandez-Miranda JC Zwagerman NT Abhinav K Lieber S Wang EW Snyderman CH. Cavernous Sinus Compartments From the Endoscopic Endonasal Approach: Anatomical Considerations and Surgical Relevance to Adenoma Surgery. J Neurosurg (2017) 129:430–41. doi: 10.3171/2017.2.JNS162214 28862552

[4] Frank G Pasquini E Farneti G Mazzatenta D Sciarretta V Grasso V. The Endoscopic *Versus* the Traditional Approach in Pituitary Surgery. Neuroendocrinology (2006) 83:240–8. doi: 10.1159/000095534 17047389

[5] Gondim JA Almeida JPC Albuquerque LAF Schops M Gomes E Ferraz T. Endoscopic Endonasal Approach for Pituitary Adenoma: Surgical Complications in 301 Patients. Pituitary (2011) 14:174–83. doi: 10.1007/s11102-010-0280-1 21181278

[6] Jang JH Kim KH Lee YM Kim JS Kim YZ. Surgical Results of Pure Endoscopic Endonasal Transsphenoidal Surgery for 331 Pituitary Adenomas: A 15-Year Experience From a Single Institution. World Neurosurg (2016) 96:545–55. doi: 10.1016/j.wneu.2016.09.051 27663264

[7] Lampropoulos KI Samonis G Nomikos P. Factors Influencing the Outcome of Microsurgical Transsphenoidal Surgery for Pituitary Adenomas: A Study on 184 Patients. Hormones (Athens) (2013) 12:254–64. doi: 10.14310/horm.2002.1409 23933694

[8] Liu J Li C Xiao Q Gan C Chen X Sun W. Comparison of Pituitary Adenomas in Elderly and Younger Adults: Clinical Characteristics, Surgical Outcomes, and Prognosis. J Am Geriatr Soc (2015) 63:1924–30. doi: 10.1111/jgs.13590 26313332

[9] Zhan R Ma Z Wang D Li X. Pure Endoscopic Endonasal Transsphenoidal Approach for Nonfunctioning Pituitary Adenomas in the Elderly: Surgical Outcomes and Complications in 158 Patients. World Neurosurg (2015) 84:1572–8. doi: 10.1016/j.wneu.2015.08.035 26341428

[10] Jolesz FA Blumenfeld SM. Interventional Use of Magnetic Resonance Imaging. Magn Reson Q (1994) 10:85–96. 7986703

[11] Bohinski RJ Warnick RE Gaskill-Shipley MF Zuccarello M van Loveren HR Kormos DW. Intraoperative Magnetic Resonance Imaging to Determine the Extent of Resection of Pituitary Macroadenomas During Transsphenoidal Microsurgery. Neurosurgery (2001) 49:1133–43; discussion 1143–4. doi: 10.1097/00006123-200111000-00023 11846908

[12] Chittiboina P. iMRI During Transsphenoidal Surgery. Neurosurg Clin N Am (2017) 28:499–512. doi: 10.1016/j.nec.2017.05.005 28917279 PMC5661990

[13] Fahlbusch R Ganslandt O Buchfelder M Schott W Nimsky C. Intraoperative Magnetic Resonance Imaging During Transsphenoidal Surgery. J Neurosurg (2001) 95:381–90. doi: 10.3171/jns.2001.95.3.0381 11565857

[14] Fomekong E Duprez T Docquier M-A Ntsambi G Maiter D Raftopoulos C. Intraoperative 3t MRI for Pituitary Macroadenoma Resection: Initial Experience in 73 Consecutive Patients. Clin Neurol Neurosurg (2014) 126:143–9. doi: 10.1016/j.clineuro.2014.09.001 25255158

[15] Gerlach R du Mesnil de Rochemont R Gasser T Marquardt G Reusch J Imoehl L. Feasibility of Polestar N20, an Ultra-Low-Field Intraoperative Magnetic Resonance Imaging System in Resection Control of Pituitary Macroadenomas: Lessons Learned From the First 40 Cases. Neurosurgery (2008) 63:272–84; discussion 284–5. doi: 10.1227/01.NEU.0000312362.63693.78 18797357

[16] Li J Cong Z Ji X Wang X Hu Z Jia Y. Application of Intraoperative Magnetic Resonance Imaging in Large Invasive Pituitary Adenoma Surgery. Asian J Surg (2015) 38:168–73. doi: 10.1016/j.asjsur.2015.03.001 25979649

[17] Makary M Chiocca EA Erminy N Antor M Bergese SD Abdel-Rasoul M. Clinical and Economic Outcomes of Low-Field Intraoperative MRI-Guided Tumor Resection Neurosurgery. J Magn Reson Imaging (2011) 34:1022–30. doi: 10.1002/jmri.22739 22002753

[18] Martin CH Schwartz R Jolesz F Black PM. Transsphenoidal Resection of Pituitary Adenomas in an Intraoperative MRI Unit. Pituitary (1999) 2:155–62. doi: 10.1023/a:1009943700810 11081166

[19] Ramm-Pettersen J Berg-Johnsen J Hol PK Roy S Bollerslev J Schreiner T. Intra-Operative MRI Facilitates Tumour Resection During Trans-Sphenoidal Surgery for Pituitary Adenomas. Acta Neurochir (Wien) (2011) 153:1367–73. doi: 10.1007/s00701-011-1004-7 PMC3111601 21523361

[20] Sylvester PT Evans JA Zipfel GJ Chole RA Uppaluri R Haughey BH. Combined High-Field Intraoperative Magnetic Resonance Imaging and Endoscopy Increase Extent of Resection and Progression-Free Survival for Pituitary Adenomas. Pituitary (2015) 18:72–85. doi: 10.1007/s11102-014-0560-2 24599833 PMC4161669

[21] Szerlip NJ Zhang Y-C Placantonakis DG Goldman M Colevas KB Rubin DG. Transsphenoidal Resection of Sellar Tumors Using High-Field Intraoperative Magnetic Resonance Imaging. Skull Base (2011) 21:223–32. doi: 10.1055/s-0031-1277262 PMC3312115 22470265

[22] Micko ASG Wöhrer A Wolfsberger S Knosp E. Invasion of the Cavernous Sinus Space in Pituitary Adenomas: Endoscopic Verification and Its Correlation With an MRI-Based Classification. J Neurosurg (2015) 122:803–11. doi: 10.3171/2014.12.JNS141083 25658782

[23] Kassam A Snyderman CH Mintz A Gardner P Carrau RL. Expanded Endonasal Approach: The Rostrocaudal Axis. Part I. Crista Galli to the Sella Turcica. Neurosurg Focus (2005) 19:E3. doi: 10.3171/foc.2005.19.1.4 16078817

[24] Dallapiazza RF Grober Y Starke RM Laws ER Jane JA. Long-Term Results of Endonasal Endoscopic Transsphenoidal Resection of Nonfunctioning Pituitary Macroadenomas. Neurosurgery (2015) 76:42–52; discussion 52–3. doi: 10.1227/NEU.0000000000000563 25255271

[25] Przybylowski CJ Dallapiazza RF Williams BJ Pomeraniec IJ Xu Z Payne SC. Primary *Versus* Revision Transsphenoidal Resection for Nonfunctioning Pituitary Macroadenomas: Matched Cohort Study. J Neurosurg (2017) 126:889–96. doi: 10.3171/2016.3.JNS152735 27203142

[26] Knosp E Steiner E Kitz K Matula C. Pituitary Adenomas With Invasion of the Cavernous Sinus Space: A Magnetic Resonance Imaging Classification Compared With Surgical Findings. Neurosurgery (1993) 33:610–7; discussion 617–8. doi: 10.1227/00006123-199310000-00008 8232800

[27] Ceylan S Anik I Cabuk B Caklili M Anik Y. Extension Pathways of Pituitary Adenomas With Cavernous Sinus Involvement and Its Surgical Approaches. World Neurosurg (2019) 127:e986–95. doi: 10.1016/j.wneu.2019.04.013 30965171

[28] Cohen-Cohen S Gardner PA Alves-Belo JT Truong HQ Snyderman CH Wang EW. The Medial Wall of the Cavernous Sinus. Part 2: Selective Medial Wall Resection in 50 Pituitary Adenoma Patients. J Neurosurg (2018) 131:1–10. doi: 10.3171/2018.5.JNS18595 30192191

[29] Truong HQ Lieber S Najera E Alves-Belo JT Gardner PA Fernandez-Miranda JC. The Medial Wall of the Cavernous Sinus. Part 1: Surgical Anatomy, Ligaments, and Surgical Technique for its Mobilization and/or Resection. J Neurosurg (2018) 131:1–9. doi: 10.3171/2018.3.JNS18596 30192192

